# Health worker motivation in the context of HIV care and treatment challenges in Mbeya Region, Tanzania: A qualitative study

**DOI:** 10.1186/1472-6963-11-266

**Published:** 2011-10-12

**Authors:** Deogratius Mbilinyi, Marguerite L Daniel, Gro Th Lie

**Affiliations:** 1 NIMR, PO Box 583 Tukuyu, Mbeya, Tanzania; 2 Department of Health Promotion and Development, University of Bergen, 5015 Bergen, Norway

## Abstract

**Background:**

Health worker motivation can potentially affect the provision of health services. The HIV pandemic has placed additional strain on health service provision through the extra burden of increased testing and counselling, treating opportunistic infections and providing antiretroviral treatment. The aim of this paper is to explore the challenges generated by HIV care and treatment and their impact on health worker motivation in Mbeya Region, Tanzania.

**Methods:**

Thirty in-depth interviews were conducted with health workers across the range of health care professions in health facilities in two high HIV-prevalence districts of Mbeya Region, Tanzania. A qualitative framework analysis was adopted for data analysis.

**Results:**

The negative impact of HIV-related challenges on health worker motivation was confirmed by this study. Training seminars and workshops related to HIV contributed to the shortage of health workers in the facilities. Lower status workers were frequently excluded from training and were more severely affected by the consequent increase in workload as seminars were usually attended by higher status professionals who controlled access. Constant and consistent complaints by clients have undermined health workers' expectations of trust and recognition. Health workers were forced to take responsibility for dealing with problems arising from organisational inefficiencies within the health system.

**Conclusion:**

HIV-related challenges undermine motivation among health workers in Mbeya, Tanzania with the burden falling most heavily on lower status workers. Strained relations between health workers and the community they serve, further undermine motivation of health workers.

## Background

Health worker motivation can potentially affect the provision of health services. Low morale among the workforce can undermine the quality of service provision and drive workers away from the profession. "The quality of the health services, their efficacy, efficiency, accessibility and viability depend on the performance of those who deliver them" [1: p.5] and it is therefore important to make personnel development a central issue in health policy. The HIV pandemic has placed additional strain on health service provision through the extra burden of increased testing and counselling, treating opportunistic infections and providing antiretroviral treatment (ART). Health workers, too, have been affected and infected by HIV adding further stress to the provision of health services.

The high level of donor attention indicates that HIV continues to be an important public health challenge in developing countries, including Tanzania [2]. In 2007 the HIV prevalence for adults (15 - 49 years) in Tanzania was 6.2 percent [3] while the figure for Mbeya, consistently higher than the national average, was 9 percent in 2008 [4]. Urban rates of HIV infection have consistently been higher than rural rates, 9 and 5 percent respectively in 2008 [4]. The government launched its first National HIV policy in 2001 - the Tanzanian Commission for AIDS was established in 2000, prior to that the HIV effort consisted of a series of plans which the Ministry of Health co-ordinated - and the antiretroviral treatment (ART) programme in 2004 [5]. By 2007 there were 204 sites providing ART to 136,000 people out of an estimated 440,000 who need treatment giving an estimated antiretroviral coverage of 31 percent, up from 14 percent in 2006 and 5 percent in 2005 [3].

There is a substantial literature on how organisational and structural factors like a shortage of health care workers can affect quality of care and health worker motivation. Tanzania experiences a severe shortage of health care workers with Munga et al. [6] claiming it has the "lowest physician/population ratio in the world". Several other studies confirm the shortage of health professionals in Tanzania [7-10] and Clemens & Pettersson [11] estimate that in 2000, 52 percent of physicians born in Tanzania were working aboard, mainly in the UK, the US and Canada. Aside from emigration, there are a number of other factors contributing to the shortage of health workers. Several studies mention HIV as a cause of health worker absenteeism and attrition [6,12,13]. Rolfe et al [7] note that in the 1990s there was a government imposed employment freeze in the health sector and those employed before then are now retiring causing high losses.

The quality and effectiveness of ART provision will be significantly affected by a country's shortage of adequate human resources for health [13]. The impact of understaffing is that one person has to do the tasks of two or three people, including those of higher status workers [8]. Schneider et al. [14: p.21] conclude "[w]ithout strengthened or even transformed health systems it is hard to see how access to ARVs can be sustainably achieved in countries with weak health systems."

The high burden of HIV and the rapid scale-up of ART increases demand for health workers significantly [6]. In the study by Rolfe et al [7] community focus groups indicated that citizens of Tanzania still strongly believe that they have a right to health care. The community has been sensitised by the government and donor community to free access to HIV testing and counselling services, and free health care for people living with HIV [15]. This may have raised expectations among community members although demand may be constrained by stigma and affordability [14].

Besides the shortage of health workers, organisational structure also refers to supply of drugs, availability of equipment and the management of health workers. Poor physical infrastructure and shortage of equipment, by reducing the quality of care, undermine motivation [8,16]. Leshabari et al. [10] found that 38 percent of the workers in their study noted that facilities were deficient for task completion and that prescribed drugs were out of stock. Manongi et al. [8] found that while some health workers received no written or oral feedback from supervision, others more often received negative rather than positive assessments. Several studies commented on the desire among health workers for further training [8,10].

While structural and organisational determinants of motivation have been widely addressed, much less has been said about other determinants. Individual level determinants describe health worker motivation as an internal psychological process involving beliefs, norms, values and attitudes that enable an individual to conceptualise appropriate behaviour [17,18]. The socio-cultural environment refers to the nature of health worker-client relationships and interaction [19]. Health workers want to be trusted by the community, they are proud of their professional identity and appreciate recognition, though they feel this is rarely forthcoming [8]. The morale of workers may also be deeply undermined by the impact of HIV, particularly where those infected seek treatment too late and die [8].

The aim of this paper is to explore the challenges generated by HIV care and treatment and their impact on health worker motivation in Mbeya Region, Tanzania. The specific objectives are to examine how HIV related challenges have affected the determinants of motivation at the individual level, at the organisational and structural level and, finally, at the level of the socio-cultural environment.

## Methods

Purposive sampling was employed to select two districts, namely Kyela and Rungwe, in Mbeya Region, Tanzania. The selection was based on HIV prevalence, availability of HIV care and treatment clinics (CTCs) as well as an adequate number of clients attending HIV CTCs. The co-ordinators of the HIV CTCs in the two districts assisted the research team in recruiting a total of 30 participants from the district hospitals (Rungwe 13, Kyela 12) and from one rural health facility in each district (Rungwe 3, Kyela 2). Recruitment purposively targeted health workers who spent a substantial amount of time in the HIV CTCs.

The qualitative design aimed to describe HIV care and treatment challenges as perceived by health workers in the selected districts. Data were collected through semi-structured interviews conducted in an office on the hospital or health centre premises. All interviews were conducted in Swahili, were recorded and were later transcribed and translated into English. The interview guide followed a number of research themes including socio-demographic characteristics, the role of the health worker, gaps in terms of skills and infrastructure, as well as job perception, experiences and recommendations.

Qualitative framework analysis was adopted as the main method for data analysis as it is well suited for applied qualitative research, particularly in health care settings [20-22]. The transcribed and translated interviews were read several times for familiarisation. The interview guide provided key themes and, as further sub-themes emerged, they were coded within the broad framework. A table was developed for each key theme and its sub-categories which, effectively, systematically mapped the data before interpretation. The quotes that have been selected are intended to represent both the central tendency in the collective responses and to show variety.

Ethical clearance for the study was obtained from the Commission for Science and Technology in Tanzania at the national level and permission to conduct the study was given by the District Medical Officers at the district level. Participation was voluntary and all participants, after receiving verbal and written information about the study, signed consent forms; they also received assurance of anonymity and confidentiality. Given that so few health centres were involved, quotes are identified only by the health care profession of the respondent to protect anonymity of individual participants.

## Results

### Socio demographic information

The distribution of the 30 participants in the study across the health care professions within the Tanzanian health services is shown in Table 1.

**Table 1:** Distribution of participants across health care professions

Health care profession	Number
Medical Officer	1
Nurse Officers	6
Clinical Officers	6
Health Officer	1
Assistant Medical Officers	2
Laboratory Technicians	3
Nurse Midwives	7
Medical Attendants	3
Nurse Attendant	1
**Total**	**30**

Some of those interviewed were also administrators or supervisors in addition to their health care role, for example, two nurse officers were also supervisors, and the medical officer was also overall coordinator of the HIV Care and Treatment Clinic (CTC). Thirteen of the participants had less than five years work experience while four had over twenty years work experience. Two thirds of the participants were women and the age range was 26 to 55 years with two thirds aged between 31 and 45 years.

### Individual level determinants

The majority of participants were attracted to join the health services by a feeling of responsibility and desire to improve people's health. However, since joining, many have developed negative attitudes or have failed to meet some of their work-related expectations.

The CTC health workers reported that they spend much of their time with people living with HIV (PLWHIV) and their perceived risk of contracting HIV and tuberculosis has contributed to a negative attitude towards their job.

*I know that I am at risk of contracting HIV because everyday I deal with people living with HIV. This affects my job attitude, I am not enjoying being a health worker anymore. - *Nurse Midwife

*This is a risky job which can expose me to contracting HIV. Imagine if I get AIDS, do you think my husband will believe that I contracted it at work? This will be a double problem to me*. - Nurse Officer

Health workers described themselves as being over-cautious about contracting HIV from their clients and noted that this sometimes affects the quality of service delivery, for example when they avoid physical contact with PLWHIV having open wounds the patients may perceive this as a form of stigma. Because many health workers originate from the communities in which they work they sometimes try to avoid being too cautious when they know the client.

Lower status workers reported that senior officers failed to acknowledge and appreciate their work, even for those with long work experience.

*I think we give everything trying to do our job, but sometimes we don't get good and supportive feedback from our senior officers who seem not to value our efforts. It is something that disappoints me very much*. - Nurse Midwife

Participating health workers from both districts, all of them female with little work experience, also experienced a lack of acknowledgement and appreciation from the community. They placed a high value on their work because they believed their job was very important, however, they felt undervalued by community members who sometimes blamed health workers for poor service at the hospital.

*This is the type of job in which you hardly get any compliments from clients and community members, rather people complain, sometimes even if you are not the source of the problem. We are always victims when things go wrong, people never think about other factors that might have caused the problem. That is why I am tired of this job*. - Nurse Midwife

### Organisation and structural level determinants

Organisational structure played an important role in motivating health workers. Essential drugs, human resources and sufficient infrastructure in the health care system all contributed to the efficient delivery of health services. Participants reported that clinics run by the district provide a comprehensive service for the PLWHIV, including voluntary counselling and testing, the provision of ART, treatment of opportunistic infections, preventing mother to child transmission and home based care. People have free access to all the services including ART once they meet the criteria, which include having a CD4 count of less than 200.

Health workers in the two districts had many additional duties not directly related to their primary job description and some senior health workers were involved in a number of other activities apart from HIV care and treatment. One assistant medical officer said,

Due to the shortage of health workers I perform several additional duties in this hospital, I normally attend patients in wards, run the CTC unit, supervise rural health centres and I am also a member of Council Health Management Team which is responsible for planning health matters at the district level. Apart from nursing knowledge, I was not trained to do any of the additional activities I am doing.

The increased number of PLWHIV has caused additional duties and pressure at work for health workers from all health care professions across the range of work experience, even in rural facilities where there is usually no special service for PLWHIV.

*I would rather work in any other department of the hospital but not here at CTC. We daily receive a lot of clients coming for clinic and we make sure that we attend all of them no matter how long it is going to take. We are kind of tied up with this job because it involves humanity issues. It's not like working in a factory*. - Clinical officer

Health workers, especially lower status workers and urban based health workers reported that they often work overtime without any pay or non financial incentives.

The shortage of drugs for opportunistic infections and lack of essential supplies were mentioned among the common problems at the hospital. This was mainly associated with an increased number of PLWHIV because, on the one hand, they use a lot of drugs and, on the other hand, they are exempt from health cost-sharing which reduces hospital revenues and hence the ability to buy drugs.

*We often run out of drugs for opportunistic infections and you know some of these drugs are very expensive; this makes our job difficult because it really annoys people living with HIV when you tell them to go and buy drugs from a local store. When they don't get some of the drugs, they don't value our service and don't see the point of coming here again. It really affects them and I personally feel bad*. - Nurse midwife

The shortage of drugs and essential supplies such as reagents for testing for HIV not only caused difficulties in treating PLWHIV, but also affected the health worker-patient relationship as well as the health workers' morale. The lack of drugs contributed to failure to treat the opportunistic infections associated with HIV infection and as a result some patients experienced a relapse and had to visit the hospital more frequently. This increased the workload and overwhelmed the health infrastructures such as wards and the laboratory.

Participants reported that HIV-related seminars and training, provided for capacity building, have actually contributed to the shortage of health workers. Seminars tend to be attended by health workers from the senior health care professions, causing them to be absent from work. This has affected the motivation of the remaining health workers because they have to work overtime without pay and the hospital has not taken any initiative to add more health workers to reduce the workload.

*We have two medical officers here but you hardly find them at all at the hospital; they are always away attending workshops and seminars related to HIV. It's just too much, they cause a heavy work load for us and sometimes we perform procedures which are strictly for them*. - Clinical officer

*We sometimes receive very complicated cases which we can't handle and since all the medical doctors are away for training and other administrative duties we just tell the patients to look for another hospital or wait until the doctors come back*. - Nurse midwife.

This was reported by health workers across the distribution of sex, age and work experience. Not surprisingly, the issue of shortage of health workers and absence due to HIV-related activities such as seminars and workshops was hardly reported by health workers from senior health care professions.

Many health workers complained that course selection processes involved some favouritism. They added that the same kind of favouritism happened when the off-duty schedules were arranged.

*Sometimes our senior officers are biased and often they favour some of our colleagues by giving them a lot of days to rest. This affects our motivation and makes our work more difficult and unattractive*. - Clinical officer

### Socio-cultural environment determinants

Participants noted that good support and cooperation from community members plays a vital role in motivating health workers, but it depends much on the quality of service received from health workers. A frequent complaint concerns the long waiting due to the increased number of clients attending the CTC, the shortage of health workers and lack of infrastructure such as consultation rooms.

*Sometimes when coming to work in the morning we find PLWHIV are already here waiting for the service, but due to our workload they sometimes wait for the service until when we are about to close the clinic, this annoys them and they start complaining to us because of long waiting*. - Nurse officer

The increased number of clients has reduced the time health workers can spend with each client, and this has led to complaints by PLWHIV that health workers do not take good care of them. Clients have sometimes claimed that health workers do not listen carefully to their problems before writing the prescription.

*We sometimes spend a very short time in attending each client in order to finish all of them, but we know that some of them are not happy with it and that is why they complain*. - Nurse midwife

Health workers reported feeling demoralised by such complaints about efforts to help their clients as there is nothing they can do to reduce the problem. This was reported by several urban based CTC health workers in the study.

Other factors mentioned as a source of complaints include lack of basic skills for attending PLWHIV and breaches of confidentiality. This was reported by urban based female health workers who have long work experience and seemed to be dissatisfied with their colleagues' performance.

*Some of health workers are not skilled enough in attending PLWHIV and this makes their job more difficult, it really affects their motivation for work because they are not skilled enough in this area of HIV*. - Nurse midwife

*Some weeks ago one person came here for the HIV test and he was confirmed positive. He had later realized that the information regarding his health status was spread all over the town and he decided to come here to the hospital and fight with a health worker he suspected of revealing the information. As a health worker I felt very bad, because we are now ruining our good reputation and losing trust and respect from our patients. Many people now who come for the HIV test are not comfortable because of not being certain with the issue of confidentiality, and some of them would rather travel to test in another district*. - Nurse officer

Almost all health workers noted that PLWHIV expect to get drugs for opportunistic infections from the hospital but hospitals have often run out of drugs and this causes a lot of complaints.

*Sometimes some of the PLWHIV use abusive language when we tell them we don't have drugs, this is because they think we don't just want to help them, something which is completely untrue. We really want to help them but we can't because of the shortage of drugs we often face*. - Clinical officer

*People living with HIV always blame us when we tell them that we don't have some of the drugs for opportunistic infections. This is because they always hear from politicians and HIV activists that there are plenty of drugs here without specifying the types of drugs. This makes our job difficult because we have to explain this over and over again and some of them they still don't believe and think that maybe we are selling drugs. These politicians they should be considerate because by not specifying they are causing us a lot of problems*. - Nurse Attendant

## Discussion

The results are presented according to the determinants of motivation: individual, structural/organisational and sociocultural; however there is significant overlap among these determinants in their impact on motivation and it is sometimes difficult to disentangle a single cause for declining motivation. To name just one example, the shortage of drugs to treat opportunistic infections (organisational) causes tension between health workers and their clients (sociocultural) leading to the perception by health workers that the community does not recognise their contribution (individual).

### HIV care and treatment

The comprehensive service for PLWHIV and the fact that they are exempt from health cost-sharing and entitled to free health care whenever they visit health facilities reflects the high level of donor attention to the implementation of HIV care and treatment services. The findings show that since the introduction of the free HIV care and treatment services in the two districts studied the perception of the health workers is that there has been an increase in the number of clients attending the clinics. This is attributed largely to heightened community awareness and raised expectations following information campaigns by government with support from donors. The districts reported benefits from donor support which helps them to provide the free service to PLWHIV and enable those who adhere to ART to continue with their daily activities.

The shortage of drugs to treat opportunistic infections was consistently mentioned by *all health care professions *as problematic, both in terms of treating patients and in terms of relations with the community. While health workers in both districts reported enough ARV drugs in their clinics, thanks to donor support, they face shortages of drugs for opportunistic infections. One reason is increased demand arising from the exemption for PLWHIV concerning health cost-sharing. In addition, the health system has failed to accommodate the increased number of PLWHIV, partly through the expectation that PLWHIV on ART can control the recurrence of infections thus reducing the need for hospital visits. However this has not been the case in the study districts as health workers reported that their clients have frequently experienced relapse of infections due to the lack of drugs for opportunistic infections. One study showed that more than 25 percent of the hospital services in Tanzania are provided to the people living with HIV [23]. Despite having comprehensive therapy in these hospitals, the failure to ensure a good supply of drugs and other essential equipment weakens the HIV care and treatment clinics in these districts.

Another factor mentioned by all health care professions was the increased workload related to people living with HIV (PLWHIV). The newly introduced HIV care and treatment services comprise a number of programs that are supposed to be managed by available health workers. Both districts have comprehensive services for PLWHIV, centralised at the district hospitals. Although existing health workers lack knowledge in attending PLWHIV, other studies suggest that donors who support HIV programs pay little attention to recruiting and training additional health workers [24-26].

Health workers reported performing several additional duties from technical to administrative tasks and supervision beyond their job specification. The shortage of health workers means that Nurse Officers and Medical Officers who should be more competent in handling HIV complications were frequently engaged in administrative and supervision duties. This, in turn, shifts additional technical duties to lower status workers such as Nurse Midwives and Clinical Officers who are less competent in the area of HIV as is also noted in the Manongi et al. [8] study. Theoretically, a worker's technical and intellectual capacity will help them to perform their assigned duties. Health workers in this study, especially the Nurse Midwives, felt that they lacked the right skills to attend PLWHIV. HIV complications associated with opportunistic infections, drug resistance, and monitoring CD4 counts, may be beyond the technical capacity of Nurse Midwives. Health workers' lack of proper skills to attend PLWHIV has been reported by other studies in Tanzania [8,26,27]. It has been noted elsewhere that the quality of staff and service provision significantly affects efficacy of ARV treatment [28,29].

Most of the other HIV-related factors affecting motivation (fear of becoming infected at work; dealing with complaints about long waiting times, short consultations, and confidentiality about status) were mentioned largely by *urban *health workers. This is to be expected as there are no special services for PLWHIV in rural areas and HIV infection rates are higher in urban than in rural areas [4]. The risk associated with taking care of PLWHIV was frequently mentioned by the interviewed health workers. Despite using protective gear health workers believe that they are still at risk of contracting HIV. Health workers face difficulties in compromising between delivering a good service and protecting themselves from contracting HIV. This is made more difficult by the fact that the majority of health workers originated from the same community as their clients. Health workers who had long working experience admitted that this is the most difficult time in their career as they joined the profession long before HIV had become a serious problem.

### Lower status health workers

The shortfall in the number of health workers needed to meet the increased demand for services has been exacerbated by various organisational practices. Firstly, the majority of respondents, besides working in CTC, have additional duties such as administration and supervision which reduce the time spent on care and treatment of PLWHIV. Secondly, this study has found that training seminars and workshops related to HIV contribute to the shortage of health workers in the facilities. Typically such seminars involve payment for attendance and it is senior officers who usually participate leaving the clinics in the hands of lower status and less experienced health workers. This introduces frustration among the lower status workers as the majority lack the opportunity to upgrade their skills as well as the possibility to earn extra income from allowances given during the seminars; in addition, the absence of senior officers adds to their workload. Respondents from the lower health care professions explained that these issues affected their motivation both directly and indirectly when they failed to provide a desirable service to their clients.

In fact, most of the challenges affecting motivation seem to be concentrated among the lower health care professions. They perceive other instances of unfair treatment: they work overtime with no extra pay or non-financial incentives and they experience favouritism in the allocation off-duty schedules. They also sense a lack of acknowledgement from both senior colleagues and from the community for the work they do. This was reported across the range of work experience and ages. Other studies have reported on the lack of appreciation and the fact that supervisors only pass on negative comments to lower status workers [8].

### Relations with the community

The interaction between health workers and their clients can affect health workers' motivation to work. Trust, recognition and appreciation from the community can enhance the ability and willingness of health workers to provide an efficient service. Willis-Shattuck et al. [16] note that this was a finding in 70 percent of studies they reviewed. In this study health workers described initial enthusiasm for their work and they explained that despite the difficulties that they face, they do their best to provide a good service to their clients. Yet despite donor support, HIV has put health care delivery under significant strain which has affected the relationship between client and health worker. Several studies have highlighted problems like lack of skills and shortage of drugs as obstacles in scaling up HIV treatment [30,31]. Building on similar findings, this study has shown how problems like the shortage of drugs, long waiting times, short consultation times and poor service affect the health worker-client relationship. Constant and consistent complaints have undermined health workers' expectations of trust and recognition. This situation frustrates health workers as their clients put all the blame on them regardless of whether the problem such as the shortage of drugs is beyond the health workers' control. This study shows how health workers are forced to take responsibility for dealing with problems arising from organisational inefficiencies within the health system. For instance, some clients think the shortage of drugs is caused by health workers who steal drugs, or that long waiting times and short consultations reflect health workers who do not want to take good care of them. This finding is in line with the study by Gilson et al. [32] where community focus groups "strongly criticised staff ... for being rude and lacking respect for their clients". They also commented on their "bullying tactics" and lack of care for their patients. Staff in these facilities also judged relations with the community to be poor. A study in Tanzania by Watt et al. [33] found that clients who rated relations with health workers as poor had worse adherence to ARVs. Similarly, in a study by Lindelow and Serneels [34] health workers reported many complaints from the community about poor treatment or inadequate service but saw the causes as beyond their control.

### Limitations

This study focuses on only a few HIV CTCs in one country and, being a qualitative study, the results cannot be generalised beyond the health care professionals and centres that participated. Nonetheless the issues emerging reflect phenomena relevant for HIV care in developing countries of which donors and policy makers should be aware. The two types of health care centres (district hospitals and rural health facilities) are very different in the HIV-services they offer: while district hospitals provide comprehensive treatment and care, rural health facilities are more limited in what they can offer. This is also reflected in the range of duties, challenges and workload of health providers and hence in the numbers of health workers recruited for this study from each type of centre. The fact that the co-ordinators of the district hospitals helped recruit participants - and also requested a copy of the report - may compromise anonymity of participants.

## Conclusions

This study has presented the HIV-related challenges that affect motivation among health workers in Mbeya, Tanzania. Several aspects related to HIV care and treatment have contributed significantly to undermining motivation among health workers with the burden falling most heavily on lower status workers. Individual determinants include the fear of contracting HIV and having to perform duties beyond their capabilities. Organisational factors which undermine motivation of health workers involve issues such as the shortage of drugs to treat opportunistic infections and the fact that most of the HIV-related skills training is given to senior health care professions. Several individual and organisational factors have an impact on the sociocultural environment leading to strained relations between health workers and the community they serve, further undermining motivation of health workers. Health workers from lower health care professions are under more pressure than their senior colleagues.

## Abbreviations

ART: antiretroviral treatment; CTC: care and treatment clinic; PLWHIV: people living with HIV.

## Competing interests

The authors declare that they have no competing interests.

## Authors' contributions

DM designed the study, acquired the data and was responsible for the analysis and interpretation of the data as part of his Master's degree. MD wrote the study in its current form as an article. GL, as supervisor to DM, contributed to the design, analysis and interpretation. All the authors read and approved the final manuscript.

## Pre-publication history

The pre-publication history for this paper can be accessed here:

http://www.biomedcentral.com/1472-6963/11/266/prepub
